# Stable QTLs for Plant Height on Chromosome A09 Identified From Two Mapping Populations in Peanut (*Arachis hypogaea* L.)

**DOI:** 10.3389/fpls.2018.00684

**Published:** 2018-05-25

**Authors:** Jianwei Lv, Nian Liu, Jianbin Guo, Zhijun Xu, Xinping Li, Zhendong Li, Huaiyong Luo, Xiaoping Ren, Li Huang, Xiaojing Zhou, Yuning Chen, Weigang Chen, Yong Lei, Jinxing Tu, Huifang Jiang, Boshou Liao

**Affiliations:** ^1^Key Laboratory of Biology and Genetic Improvement of Oil Crops, Ministry of Agriculture, Oil Crops Research Institute of the Chinese Academy of Agricultural Sciences, Wuhan, China; ^2^National Key Laboratory of Crop Genetic Improvement, National Sub-Center of Rapeseed Improvement in Wuhan, Huazhong Agricultural University, Wuhan, China; ^3^Guizhou Oil Research Institute, Guizhou Academy of Agricultural Sciences, Guiyang, China

**Keywords:** QTL analysis, plant height, meta-analysis, cultivated peanut, RIL population

## Abstract

The peanut (*Arachis hypogaea* L.) is an important grain legume extensively cultivated worldwide, supplying edible oil and protein for human consumption. As in many other crops, plant height is a crucial factor in determining peanut architecture traits and has a unique effect on resistance to lodging and efficiency of mechanized harvesting as well as yield. Currently, the genetic basis underlying plant height remains unclear in peanut, which have hampered marker-assisted selection in breeding. In this study, we conducted a quantitative trait locus (QTL) analysis for peanut plant height by using two recombinant inbred line (RIL) populations including “Yuanza 9102 × Xuzhou 68-4 (YX)” and “Xuhua 13 × Zhonghua 6 (XZ)”. In the YX population, 38 QTLs including 10 major QTLs from 9 chromosomes were detected in 4 environments, and 8 consensus QTLs integrated by meta-analysis expressed stably across multiple environments. In the XZ population, 3 major QTLs and seven minor QTLs from 6 chromosomes were detected across 3 environments. Generally, most major QTLs from the two populations were located on pseudomolecule chromosome 9 of *Arachis duranesis* (A09), indicating there would be key genes on A09 controlling plant height. Further analysis revealed that *qPHA09.1a* from the XZ population and one consensus QTL, *cqPHA09.d* from the YX population were co-localized in a reliable 3.4 Mb physical interval on A09, which harbored 161 genes including transcription factors and enzymes related to signaling transduction and cell wall formation. The major and stable QTLs identified in this study may be useful for further gene cloning and identification of molecular markers applicable for breeding.

## Introduction

The cultivated peanut or groundnut (*Arachis hypogea* L.) is one of the most important oilseed and cash crops worldwide and is a crucial source of edible oil and protein for human consumption. It is widely cultivated in several tropical and sub-tropical regions, with a global harvest area of 26.54 million ha and a production of 42.32 million tons (FAOSTAT, 2014). Currently, China, India and the USA are among the top peanut producing countries in the world. The peanut production in China in 2015 was 16.44 million ton, ranking the first in the world and the first among domestic oil crops in China (http://zzys.agri.gov.cn/nongqing.aspx). For most crops, plant height is an important architecture trait largely affecting photosynthesis efficiency and resistance to lodging (Falster and Westoby, 2003; Salas Fernandez et al., 2009; Sarlikioti et al., 2011). Previous studies have shown a statistically significant correlation between plant height and yield-related traits in peanut (Jiang et al., 2014; Huang et al., 2015). In addition, lodging due to too long of a main stem could reduce yield and make the mechanized harvest of peanuts more difficult. The aim in peanut breeding is therefore cultivation of varieties with desirable plant height that facilitates mechanized harvest and increases final yield. Thus, understanding the genetic inheritance pattern of plant height is key to a knowledge-based improvement of plant height.

Quantitative trait locus (QTL) analysis is a useful approach to dissect the complicate quantitative trait, and dozen of additive and epistatic QTLs for plant height have been identified in major cereal crops (Zhang et al., 2006, 2017; Wu et al., 2010; Cui et al., 2011; Lee et al., 2014; Han et al., 2017). Of them, major genes/loci such as *Rht-B1b* and *Rht-D1b* in wheat, and *sd1* in rice were well characterized and widely used in breeding programs (Peng et al., 1999; Sasaki et al., 2002; Asano et al., 2007; Würschum et al., 2015). Map-based cloning and functional analyses were shown that several QTL genes involve in biogenesis or signal transduction of gibberellin acid, brassinosteroids and strigolactones to regulate plant height (Ikeda et al., 2001; Sasaki et al., 2002; Zou et al., 2005; Tong et al., 2012; Teng et al., 2013; Wilhelm et al., 2013). As to peanut, the genetic basis of controlling plant height remains currently unclear, although there is a great diversity in the plant height of germplasm collections of both cultivated species and wild *Arachis* accessions.

Currently, many QTL mapping studies using bi-parental population have been conducted to identify QTLs for pod- or seed-related traits, oil quality, and resistance to biotic stresses such as rust, late leaf spot and *Meloidogyne arenaria* in peanut (Pandey et al., 2014, 2016; Varshney et al., 2014; Leal-Bertioli et al., 2015; Chen et al., 2016; Zhou et al., 2016; Luo et al., 2017a,b). While limited efforts have been made to detect QTLs associated with plant height in peanut. Shirasawa et al. (2012) first identified 3 QTLs with 4.8–19.2% phenotypic variation explained for plant height in 94 F_2_ lines. Similarly, another three QTLs with interval distances of 8.1–16.8 cM were identified based on an F_2:3_ mapping population (Huang et al., 2015). However, all QTLs from these two reports were detected in a single environment. More recently, our lab reported 18 QTLs for plant height in an RIL population and found that two consensus QTLs on linkage group (LG) A04 performed stably across environments (Huang et al., 2016). Li et al. (2017) subsequently identified three other consistently expressed QTLs with interval distances of 5.97–6.71 cM across multiple environments in a single RIL population.

From previous studies, only a few QTLs with stable performance across environments were identified. However, no major QTLs detected in one specific population could be valid in other populations with different genetic backgrounds indicating that these QTLs were less meaningful in peanut breeding. To overcome this problem, two peanut RIL populations were constructed and used in this study to identify robust QTLs controlling plant height with stable performance across multiple environments and explore their potential in marker-assisted selection breeding.

## Materials and methods

### Plant materials and field trials

Two RIL populations were developed from two crosses, Yuanza 9102 × Xuzhou 68-4 and Xuhua 13 × Zhonghua 6, through the single seed decent method. Yuanza 9102, the female parent of the YX population, belonging to *A. hypogaea* subsp. *fastigiata* var. *vulgaris*, was derived from interspecific hybridization between cultivated cultivar Baisha 1016 and a diploid wild species *A. diogoi*. Xuzhou 68-4, the male parent, which belongs to *A. hypogaea* subsp. *hypogaea* var. *hypogaea*, had significantly higher plant height than the female parent Yuanza 9102. Xuhua 13, the female parent of the XZ population, belongs to *A. hypogaea* subsp. *hypogaea* var. *hypogaea*. Zhonghua 6, the male parent of the XZ population, belongs to *A. hypogaea* subsp. *fastigiata* var. *vulgaris*. The height of Xuhua 13 is slightly higher than Zhonghua 6.

Two mapping populations, consisting of 195 (YX) and 188 (XZ) lines, were used to generate genotypic data for QTL analysis in this study. The two populations with their parents were planted in the experimental station of OCRI-CAAS, Wuhan, China. For the YX population, the trials of four consecutive years from 2013 to 2016 were named as YX2013, YX2014, YX2015, and YX2016. For the XZ population, the trials from 2014 to 2016 were named as XZ2014, XZ2015, and XZ2016. Field trials were performed on a randomized complete block design with three replications. Each plot contained 12 plants in one row, with 20 cm between plants and 30 cm between rows. According to a described standard method, at least 8 of the 12 plants were selected to record plant height through measuring the length from base of the above-ground plant to the tip of the main stem (Huang et al., 2016). All field management followed standard agricultural practices.

### Statistical analysis of phenotypic data

The phenotypic data of plant height trait was analyzed by IBM SPASS Statistics software (2013). The Shapiro-Wilk test was used to assess the normal distribution of phenotypic data in each year. The univariate variance analysis was performed through standard GLM method, and restricted maximum likelihood method was used to evaluate variance components. The broad-sense heritability was calculated based on the following formula: $\;H^{2}=\;\sigma_{g}^{2}/\left(\sigma_{g}^{2}+\sigma_{g\times e}^{2}/r+\sigma_{e}^{2}/rn\right)$, where $\sigma_{g}^{2}$ is the genotypic variance among RILs, $\sigma_{g\times e}^{2}$ is the variance of interaction between genotype and environment, $\sigma_{e}^{2}$ is the residual variance, r is the number of trial environments and n is the number of replications in each environment (Holl et al., 2010).

### QTL mapping and meta-analysis

Genome-wide QTL mapping was performed using the composite interval mapping (CIM) method through QTL Cartographer 2.5 software (Zeng, 1994). By 1,000 times permutation with *P* < 0.05, the LOD thresholds for plant height were 3.5, 3.3, 3.2, and 3.4 in the 2013–2016 trials of YX population, and 3.4, 3.4, and 3.3 in 2014–2016 trials of the XZ population. The walk speed, control marker and window size were set as 1, 5, and 10 cM respectively. The QTLs which had phenotypic variation explained more than 10% were considered as major QTLs, otherwise considered as minor QTLs. According to previously described nomenclature (Udall et al., 2006), QTLs were designated with an initial letter “*q*” followed by the abbreviation of trait name “*PH*” and the corresponding linkage group. After linkage group, codes 1, 2, 3, and 4 were added representing the 2013, 2014, 2015, and 2016 trials of the YX population respectively, and codes 1, 2, and 3 were added after linkage group representing 2014, 2015, and 2016 trials of XZ population respectively. If more than one QTL was identified in the same linkage group and same year, an alphabetical letter was added after the code. For instance, if two QTLs associated with plant height were identified on LGA09 of YX population in 2013, they were named as *qPHA09.1a* and *qPHA09.1b*.

QTLs identified from different environments but located in the same linkage were subjected to the meta analysis to estimate the position of consistent QTL through BioMercator 2.1 software (Goffinet and Gerber, 2000; Arcade et al., 2004). The consistent QTLs were designated with an initial letter “c*q*” followed by the trait name and linkage group. An alphabetical letter was added after the linkage group if more than one consistent QTL was located there.

### Gene annotation in co-localized region

Through blast search, markers linked to QTLs were located in the genome of diploid species which are regarded as the ancestors of the cultivated peanut (Bertioli et al., 2016). The corresponding genes sequence and transcript abundance were downloaded from PeanutBase (Bertioli et al., 2016) and *Arachis* eFP Browser (Clevenger et al., 2016), respectively. GO analysis and KEGG analysis were performed using the software Blast2GO (Conesa et al., 2005).

## Results

### Phenotypic variation in plant height

**Figure 1:**
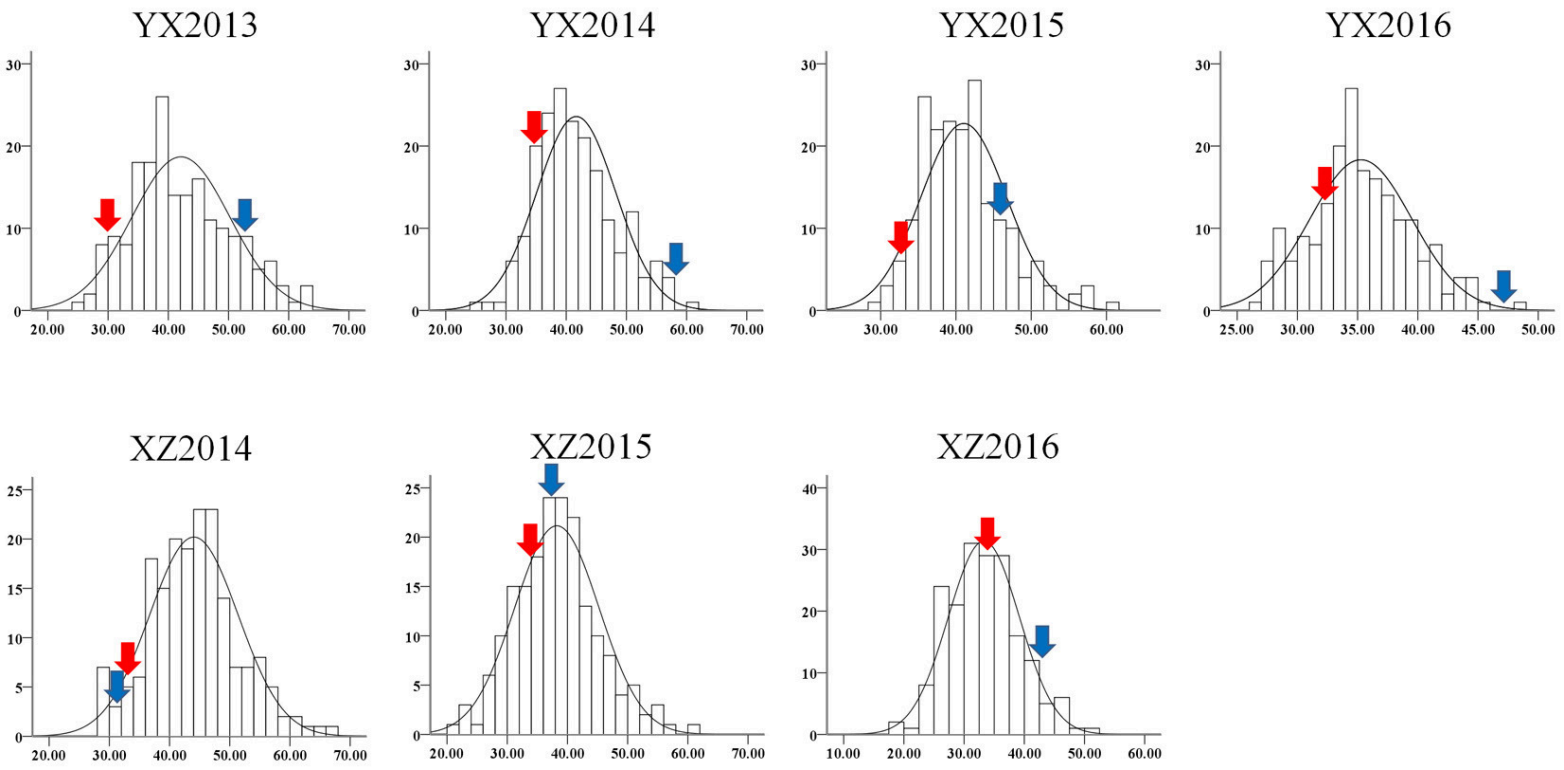
Phenotype distribution of plant height in two RIL populations. The y-axis represents frequency; the x-axis represents value of plant height. YX “Yuanza9102 × Xuzhou 68-4” RIL population, XZ “Xuhua 13 × Zhonghua 6” RIL population. Red and blue arrows denote female and male parents, respectively.

The plant height trait was recorded in both the YX and XZ populations. In the YX population, a significant difference was found between the two parental genotypes and among the RILs (Table 1). The plant height of the female parent (Yuanza 9102) ranged from 29.22 to 34.57 cm, whereas the height of the male parent (Xuzhou 68-4) varied from 46.85 to 58.14 cm in four environments. The phenotypic variation in the RILs varied from 25.80 to 74.38, 25.80 to 61.50, 28.40 to 60.30, and 26.70 to 48.70 cm in the four environments. A significant variation was also observed in the RILs of the XZ population across three environments (Table 1), ranging from 28.00 to 66.70, 21.86 to 61.54, and 17.68 to 51.41 cm. However, the plant heights of the two parents (Xuhua 13 and Zhonghua 6) in the XZ population were similar across multiple environments. Generally, the phenotypic data for both populations showed continuous distributions with transgressive segregation (Figure 1). The Shapiro-Wilk (*w*) normality test further indicated that phenotypic data from the XZ population across multiple environments and phenotypic data from the YX population in 2016 were normally distributed (Table 1). Two-way analysis of variance revealed that genetic and environmental factors could significantly influence plant height in both populations, and genotype × environment interactions could also significantly influence the phenotype in the YX population (Table 2). The broad sense heritability of plant height was estimated to be 0.81 for the YX population and 0.89 for the XZ population, indicating plant height was mainly controlled by genetic factors in both populations.

**Table 1:** Descriptive statistical analysis for plant height (cm) in two RIL populations.

**Pop**	**Env**	**P1**	**P2**	**Range(cm)**	**Mean(cm)**	**SD**	**Kurt**	**Skew**	**w(Sig)**
YX	YX2013	29.22	53.50	25.80–74.38	42.09	8.16	−0.38	0.41	0.98 (0.005)
	YX2014	34.57	58.14	25.80–61.50	41.55	6.47	0.03	0.47	0.98 (0.013)
	YX2015	32.62	46.85	28.40–60.30	41.04	5.75	0.78	0.77	0.96 (0.000)
	YX2016	32.50	47.85	26.70–48.70	35.14	4.17	0.01	0.29	0.99 (0.088)
XZ	XZ2014	32.64	31.63	28.00–66.70	44.00	7.38	0.22	0.30	0.99 (0.162)
	XZ2015	34.00	37.25	21.86–61.54	38.24	7.02	0.34	0.37	0.99 (0.192)
	XZ2016	35.83	42.38	17.68–51.41	33.29	5.91	0.07	0.26	0.99 (0.429)

*Pop, population; Env, environment; P1, female parent; P2, male parent; SD, standard deviation; Kurt, kurtosis; Skew, skewness; w, Shariro-Wilk statistic value; Sig, significance; YX, “Yuanza9102 × Xuzhou 68-4”RIL population; XZ, “Xuhua 13 × Zhonghua 6” RIL population*.

**Table 2:** Two-way ANOVA of variance for plant height in two RIL populations across multiple environments.

**Population**	**Source**	**df**	**Sum of square**	**Mean square**	***F*-value**	***P-*value**
YX	Genotype	194	31,363.47	161.67	11.35	< 0.001
	Environment	3	11,021.13	3673.71	258.00	< 0.001
	Genotype × Environment	578	17,812.32	30.82	2.16	< 0.001
	Error	585	8,329.96	14.24		
XZ	Genotype	186	31,449.29	169.08	8.61	< 0.001
	Environment	2	14,262.94	7,131.47	363.25	< 0.001
	Genotype × Environment	370	6,389.14	17.27	0.88	0.89
	Error	360	7,067.75	19.63		

*YX, “Yuanza9102 × Xuzhou 68-4” RIL population; XZ,“Xuhua 13 × Zhonghua 6” RIL population*.

### Identification of QTLs for plant height

The genetic linkage maps of the YX population and the XZ population have previously been constructed in our lab (Luo et al., 2017b,c). For the YX population, the genetic map contained 830 loci spanning 1,386.2 cM, which were assigned to 20 linkage groups designated as A01–A10 for A subgenome and B01-B10 for B subgenome. Length of LGs varied from 13.8 to 125.0 cM possessing 3–110 marker loci. For XZ population, 817 polymorphic markers were successfully mapped on 20 LGs which varied from 34.3 to 134.7 cM and contained 7 to 97 marker loci. The genetic map spanned 1756.5 cM with a map density of 2.2 cM per loci.

**Figure 2:**
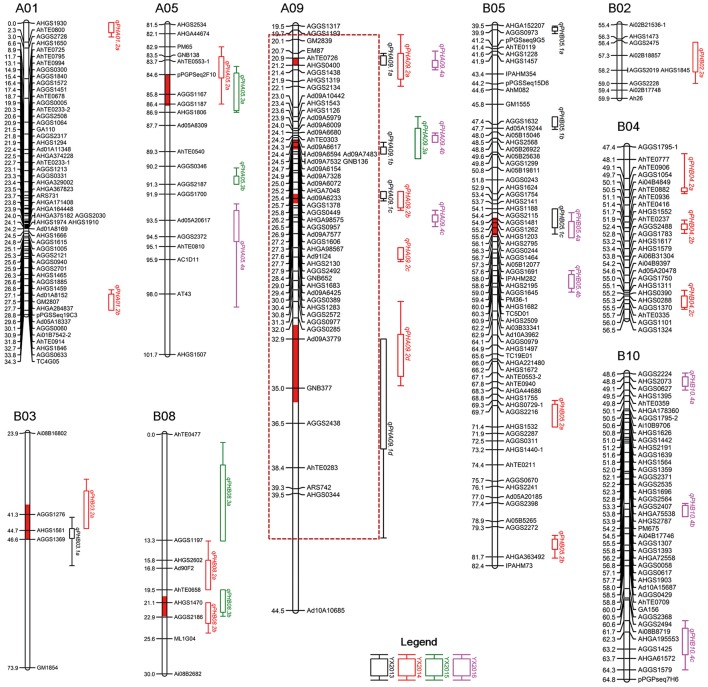
Distribution of QTLs for plant height in the genetic map of the YX population. Consistent QTLs obtained through meta-analysis are highlighted in red color on bars of linkage groups. A dashed box denotes a hot spot QTL region. YX “Yuanza9102 × Xuzhou 68-4” RIL population.

Genome-wide analyses were performed using the genetic maps and phenotypic data of plant height from RILs of the two populations. In total, 48 QTLs with 3.99–26.27% phenotypic variation explained (PVE) associated with plant height were detected in two populations across multiple environments (Table 3, Figure 2). For the YX population, eight QTLs including five major QTLs, namely, *qPHA09.1a, qPHA09.1b, qPHA09.1c, qPHA09.1d*, and *qPHB05.1b*, explaining 7.78–26.27% of the phenotypic variation, were identified in the 2013 trial. In the 2014 trial, two major QTLs, *qPHA09.2c* and *qPHB05.2b*, and 14 minor QTLs were detected with 3.99–12.85% PVEs. Only five QTLs with 4.64–9.18% PVEs were identified in the 2015 trial. In addition, six minor QTLs and three major QTLs, *qPHA09.4a, qPHA09.4b*, and *qPHB05.4b* were detected in the 2016 trial, explaining 4.83–24.74% of the phenotypic variations.

**Table 3:** QTLs of plant height were detected in two populations across multiple environments.

**Population**	**Environment**	**LG**	**QTL**	**Position(cM)**	**LOD**	**CI (cM)**	**Additive effect**	**PVE (%)**
YX	2013	A09	*qPHA09.1a*	21.01	11.27	20.70–21.20	3.68	19.67
	2013	A09	*qPHA09.1b*	24.91	16.34	24.50–25.00	4.18	25.73
	2013	A09	*qPHA09.1c*	26.91	16.72	26.60–27.00	4.21	26.27
	2013	A09	*qPHA09.1d*	33.91	11.59	32.90–41.40	3.96	23.22
	2013	B03	*qPHB03.1a*	44.71	6.89	41.90–52.20	2.48	9.13
	2013	B05	*qPHB05.1a*	39.91	5.88	39.60–40.20	2.54	8.53
	2013	B05	*qPHB05.1b*	47.41	9.12	46.80–47.80	3.30	12.49
	2013	B05	*qPHB05.1c*	54.91	5.46	54.10–56.10	2.49	7.78
	2014	A01	*qPHA01.2a*	0.01	4.51	0.00–1.70	1.51	4.90
	2014	A01	*qPHA01.2b*	32.71	3.64	31.40–33.80	1.34	3.99
	2014	A05	*qPHA05.2a*	83.71	4.42	82.90–86.40	1.48	4.84
	2014	A09	*qPHA09.2a*	21.01	6.30	19.90–22.10	1.94	8.33
	2014	A09	*qPHA09.2b*	26.91	7.09	26.60–27.40	2.04	9.32
	2014	A09	*qPHA09.2c*	29.41	7.95	29.00–29.60	2.12	10.25
	2014	A09	*qPHA09.2d*	33.91	4.52	31.30–34.90	1.93	8.17
	2014	B02	*qPHB02.2a*	57.31	3.83	56.50–59.00	1.39	4.17
	2014	B03	*qPHB03.2a*	40.91	7.79	33.80–44.30	2.15	9.96
	2014	B04	*qPHB04.2a*	49.71	4.88	47.80–49.90	1.85	6.27
	2014	B04	*qPHB04.2b*	51.71	5.42	51.30–52.00	1.95	6.79
	2014	B04	*qPHB04.2c*	55.51	4.64	55.00–56.00	1.74	5.86
	2014	B05	*qPHB05.2a*	70.81	5.27	69.30–71.40	1.85	6.92
	2014	B05	*qPHB05.2b*	80.31	7.71	80.00–81.80	2.49	12.85
	2014	B08	*qPHB08.2a*	16.81	4.19	13.40–19.50	1.51	4.68
	2014	B08	*qPHB08.2b*	22.11	4.45	21.10–24.90	1.64	5.51
	2015	A05	*qPHA05.3a*	86.41	4.50	84.10–86.90	1.54	6.88
	2015	A05	*qPHA05.3b*	91.21	4.67	90.30–91.30	1.64	7.97
	2015	A09	*qPHA09.3a*	24.51	3.08	23.40–25.20	1.29	4.64
	2015	B08	*qPHB08.3a*	9.01	4.03	1.10–13.40	1.77	9.18
	2015	B08	*qPHB08.3b*	21.11	5.85	19.50–22.80	1.76	8.96
	2016	A05	*qPHA05.4a*	93.51	3.83	92.50–98.80	0.97	5.10
	2016	A09	*qPHA09.4a*	21.21	13.15	20.60–21.40	2.03	22.28
	2016	A09	*qPHA09.4b*	24.41	14.97	24.10–24.50	2.13	24.74
	2016	A09	*qPHA09.4c*	27.71	4.26	27.40–28.10	1.41	6.23
	2016	B05	*qPHB05.4a*	56.11	6.03	54.40–56.40	1.35	8.37
	2016	B05	*qPHB05.4b*	60.01	7.57	59.30–60.70	1.45	10.31
	2016	B10	*qPHB10.4a*	49.11	3.59	48.60–49.50	−0.94	4.83
	2016	B10	*qPHB10.4b*	55.81	4.50	55.50–56.20	−1.05	5.96
	2016	B10	*qPHB10.4c*	62.31	3.67	61.70–64.30	−0.96	4.92
XZ	2014	A09	*qPHA09.1a*	34.91	3.73	29.80–36.10	2.04	7.52
	2014	B10	*qPHB10.1a*	86.61	3.42	83.20–108.20	−1.88	6.41
	2015	A05	*qPHA05.2a*	89.21	4.29	87.90–89.50	2.02	8.16
	2015	B04	*qPHB04.2a*	15.81	5.11	9.80–18.10	2.50	11.97
	2015	B04	*qPHB04.2b*	22.11	5.64	18.10–25.40	2.53	12.03
	2016	A09	*qPHA09.3a*	37.41	5.51	36.40–38.40	1.95	10.63
	2016	A09	*qPHA09.3b*	42.91	5.50	42.10–45.40	1.90	9.91
	2016	B03	*qPHB03.3a*	37.01	3.59	34.90–40.60	−1.75	8.60
	2016	B03	*qPHB03.3b*	51.31	3.88	50.90–52.30	−1.57	6.91
	2016	B08	*qPHB08.3a*	1.01	3.70	0.00–6.00	−1.54	6.73

*YX,“Yuanza9102 × Xuzhou 68-4” RIL population; XZ, “Xuhua 13 × Zhonghua 6” RIL population; LG, linkage group; LOD, logarithm of odds; CI, 2-LOD confidence interval; PVE, phenotypic variation explained*.

In total, 38 QTLs including 10 major QTLs were identified in four environments and mapped on nine LGs in YX population (Table 3, Figure 2). Of the 38 QTLs, 12 were detected in approximately 20 cM interval on LGA09, indicating there was a QTL cluster on LGA09. Interestingly, most major QTLs (7/10) with 10.25–26.27% PVE and 7.95–16.72 LOD were also located in the QTL cluster on LGA09, suggesting that LGA09 was most likely rich in key genes controlling plant height (Table 3, Figure 2). To further explore stable QTLs controlling plant height, meta-analysis was conducted to integrate QTLs detected in the four environments. Finally, eight consistent QTLs, which could be repeatedly detected in at least two environments, were identified on LGA09, LGA05, LGB03, LGB05, and LGB08 (Table 4, Figure 2). Especially on LGA09, four consistent QTLs, *cqPHA09.a, cqPHA09.b, cqPHA09.c*, and *cqPHA09.d*, located from 21.04 to 33.90 cM, in order, explained 8.33–22.28, 4.64–25.73, 9.32–26.27, and 8.17–23.22% of the phenotypic variation, respectively. In addition, four other consistent QTLs, *cqPHA05, cpPHB03, cpPHB05* and *cpPHB08*, explained 4.84–6.88, 9.13–9.96, 7.78–8.37, and 5.51–8.96% phenotypic variation, respectively.

**Table 4:** Consensus QTLs of plant height through meta-analysis in multiple environments.

**Consensus QTL**	**LG**	**Position (cM)**	**CI (cM)**	**Consistent QTLs**
*cqPHA05*	A05	85.54	84.55–86.53	*qPHA05.2a, qPHA05.3a*
*cqPHA09.a*	A09	21.04	20.87–21.22	*qPHA09.1a, qPHA09.2a, qPHA09.4a*
*cqPHA09.b*	A09	24.60	24.44–24.75	*qPHA09.1b, qPHA09.3a, qPHA09.4b*
*cqPHA09.c*	A09	26.91	26.73–27.08	*qPHA09.1c, qPHA09.2b*
*cqPHA09.d*	A09	33.90	32.25–35.56	*qPHA09.1d, qPHA09.2d*
*cqPHB03*	B03	42.84	39.17–46.52	*qPHB03.1a, qPHB03.2a*
*cqPHB05*	B05	55.51	54.80–56.21	*qPHB05.1c, qPHB05.4a*
*cqPHB08*	B08	21.53	20.29–22.78	*qPHB08.2b, qPHB08.3b*

*LG, linkage group; CI, confidence interval*.

**Figure 3:**
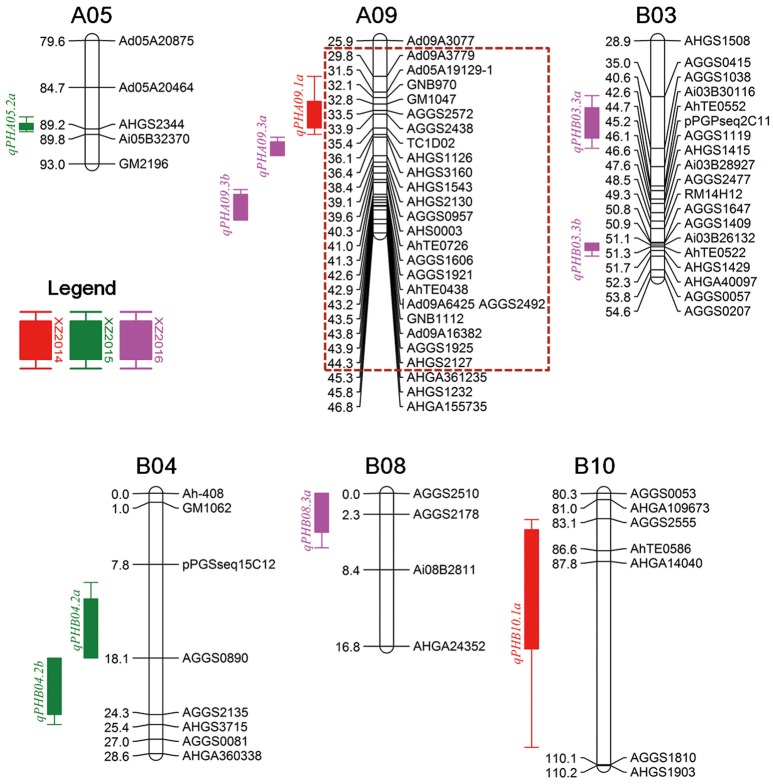
Distribution of QTLs for plant height in the genetic map of XZ population. Dashed box denoted hot spot QTL region. XZ “Xuhua 13 × Zhonghua 6” RIL population.

For the XZ population, 10 QTLs including three major QTLs with more than 10% PVE were detected in three environments (Table 3, Figure 3). In the 2014 trial, two QTLs, namely, *qPHA09.1a* and *qPHB10.1a*, were identified, explaining 6.41–7.52% of the phenotypic variation. In the 2015 trial, three QTLs, including two major QTLs, *qPHB04.2a* and *qPHB04.2b*, and a minor QTL, namely, *qPHA05.2a*, were detected with a range of 8.16–12.03% PVE. Four minor QTLs namely, *qPHA09.3b, qPHB03.3a, qPHB03.3b* and *qPHB08.3a*, and a major QTL, *qPHA09.3a*, were identified in the 2016 trial, explaining 6.73–10.63% PVE. In total, 10 QTLs were located on six LGs. Three QTLs were located in approximately 15 cM interval on LGA09 with 7.52–10.63% PVE, indicating this region may harbor genes in controlling plant height. In addition, six QTLs from three LGs had positive additive effects, indicating that the alleles increasing plant height are from male parent (Zhonghua 6). However, four other QTLs had negative additive effects, which demonstrated that the female parent also had alleles for increasing plant height. These loci controlling plant height could be recombined in the progeny. Thus, it was observed that parents did not differ much in terms of plant height, but the phenotypic data of the RIL population (XZ) was significantly different (Table 1).

### A hot spot of QTLs for plant height on chromosome A09

Comparing the QTLs identified in the two populations, we found that LGA05, A09, B03, B04, B08, and B10 harbored QTLs in both populations (Table 3). However, common markers linked to the detected QTLs between the YX and XZ populations were only found on LGA09 (Supplemental Figure 1). In the YX population, there was a QTL cluster, which could be integrated into four repeated detectable QTLs on LGA09 covering an approximately 20 cM interval. For the XZ population, LGA09 also harbored three QTLs in a 15 cM interval. In total, there were 11 common markers, *AhTE0726, AHGS1543, AHGS1126, AGGS0957, AGGS1606, AHGS2130, AGGS2492, Ad09A6425, AGGS2572, Ad09A3779*, and *AGGS2438*, between the two intervals on LGA09 from different genetic maps, indicating that there is a hot spot of QTLs for plant height on chromosome A09. Then we produced an integrated map of LGA09, and found that QTLs for plant height from the YX and XZ population were co-localized in the same interval in the integrated map, further verifying that chromosome A09 is rich in genes controlling plant height (Supplemental Figure 1).

**Figure 4:**
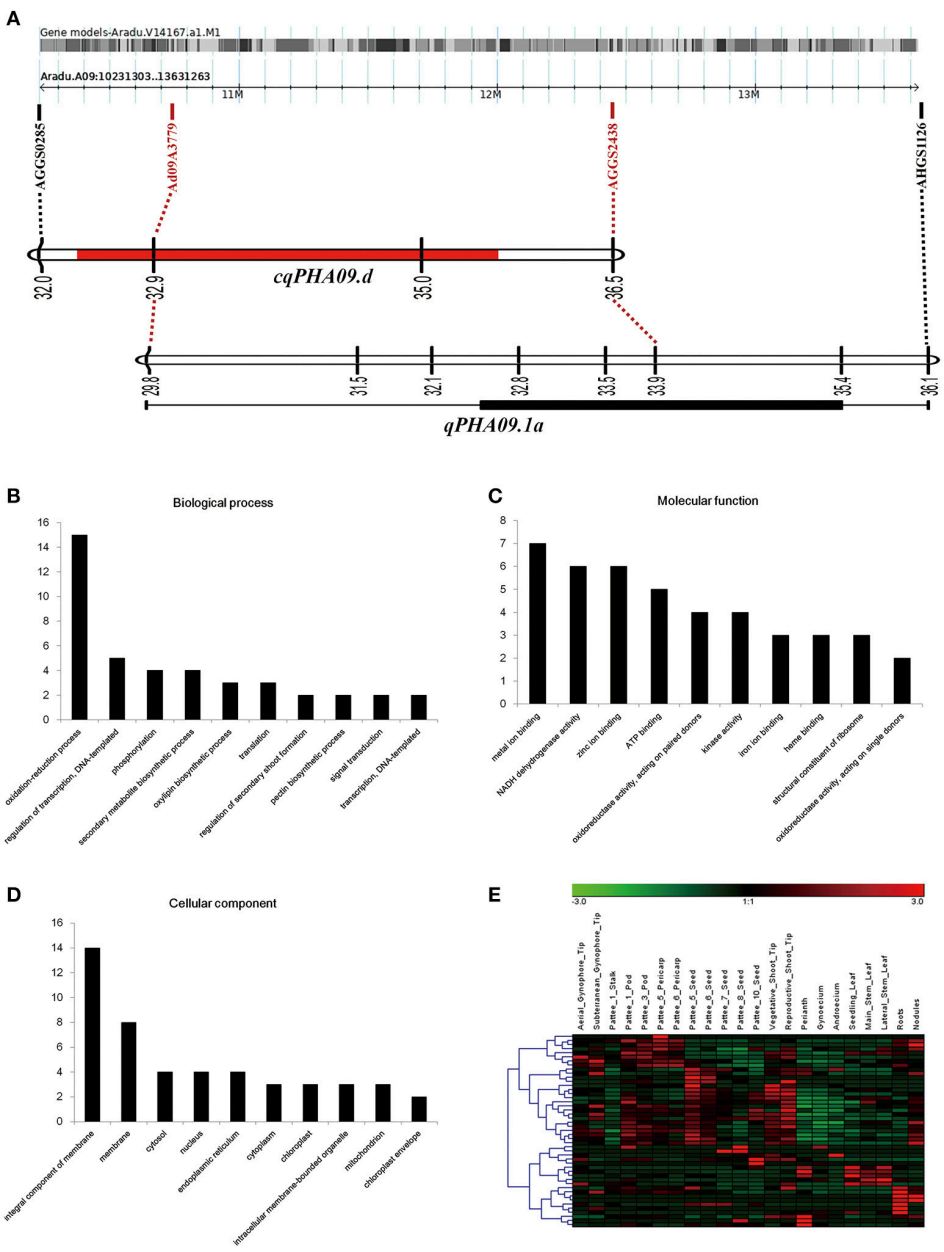
Overview of genetic and physical co-localized region on chromosome A09. **(A)** Genetic linkage groups mapping on physical chromosome A09. **(B–D)** The top ten terms of GO annotation among biological process **(B)**, molecular function **(C)** and cellular component **(D)**. **(E)** Hierarchical clustering of expression pattern in different organisms of the cultivated peanut. The values of transcript abundance were taken from (Clevenger et al., 2016).

Notably, we found that consistent QTL *cqPHA09.d* in the YX population and *qPHA09.1a* in the XZ population, had two common linked markers (Figure 4A). Through mapping markers linked to QTLs into the pseudomolecule A09 of A subgenome (*A. duranensis* V14167), 161 putative genes were detected in a 3.4 Mb physical interval (10231303–13631263 bp). One hundred and fifty genes were well annotated, whereas another 11 genes were reported to be unknown proteins, based on the results of BLAST searching for non-redundant protein sequences in NCBI (Supplemental Table S1). For GO annotation, 77 genes were assigned to at least one GO term. Among the genes involved in biological processes, oxidation reduction process and regulation of transcription were the most represented (Figure 4B). For molecular functions, metal ion binding, NADH dehydrogenase activity and zinc ion binding were the most represented (Figure 4C). Among cellular components the genes localized in, membranes were most represented (Figure 4D). Moreover, the KEGG analysis showed that 33 genes encoding oxidoreductases, transferases, hydrolases and ligases take part in 26 pathways including riboflavin metabolism, oxidative phosphorylation, biosynthesis of antibiotics, purine metabolism, amino sugar, and nucleotide sugar metabolism, etc. (Supplemental Table S2). Among all putative genes, one third (50 of 161) were found with corresponding orthologous transcripts in the transcriptome of the allotetraploid (*Arachis hypogaea* L.) (Clevenger et al., 2016). The expression pattern of these genes generally could be sorted into two subgroups (Figure 4E). The transcripts in one subgroup were mainly abundant in developmental pods, developmental seeds and tips from vegetative shoot and reproductive shoot. The members in another group were predominantly expressed in roots and nodules or leaves from seedling, main stem and lateral branch.

## Discussion

Plant height is a key trait highly related to plant architecture, resistance to lodging, biomass, yield, and adaptation to mechanized harvesting in most crops including peanut (Wang and Li, 2008; Salas Fernandez et al., 2009). In major cereal crops, well-known “green revolution” genes, *sd1* and *Rht-B1*/*Rht-D1* have been cloned, characterized and successfully applied in cultivation of semi-dwarf varieties with reduced plant height (Peng et al., 1999; Sasaki et al., 2002; Asano et al., 2007; Würschum et al., 2015). In peanut, genetic basis underlying plant height remains unclear in peanut. Since the phenotypic data of plant height is largely affected by the environment, breeders must work hard and spend time to assess the performances of varieties in multiple environments. Marker-assisted breeding has the potential to achieve higher genetic gains in less time through selecting markers linked to the QTLs for target trait (Janila et al., 2016). Several studies have been conducted in peanut to dissect the genetic natures and identify QTLs responsible for plant height; however, only single mapping population was used in the individual studies and no consistent QTLs from different populations were identified for this trait (Shirasawa et al., 2012; Huang et al., 2015, 2016; Li et al., 2017). The potential breeding value of these QTLs is thus limited because they are only effective in specific population. Therefore, it is necessry to identify consistent QTLs among different populations, which could be employed for marker-assisted breeeding in peanut.

In this study, two peanut RIL populations with different genetic backgrounds were used to explore possible consistent QTLs for plant height. Broad-sense heritability estimated in the two populations was 0.81 and 0.89 for plant height, indicating that the genetic role is dominant in controlling this trait in both populations. Using two high dense genetic linkage maps (Luo et al., 2017b) and phenotypic data from multiple environments, 38 and 10 QTLs for plant height were identified in the YX and XZ population, respectively. Through meta-analysis, 18 QTLs were integrated to eight consensus QTLs, which performed stably across multiple environments in the YX population. Previously, only three QTLs with stable performance in multiple environments were identified (Huang et al., 2016; Li et al., 2017). These stable QTLs would provide more opportunity to fine map candidate genes and further illustrate the mechanism of controlling plant height in the peanut. It is interesting to note that, one consensus QTL *cqPHA09.d* in the YX population was rightly overlapped with QTL *qPHA09.1a* in XZ population. Based on the linked markers mapped into the genome, the locus was localized in a 3.4 Mb physical region on chromosome A09. This locus could explain 23.22 and 8.17% phenotypic variations in the YX population and 7.52 phenotypic variation in the XZ population respectively. As we know, it is the first time to report a QTL for plant height which could express stably both in different populations and environments. Diagnostic markers developed from this stable QTL could be applicable in marker-assisted selection in peanut breeding.

Among the nine LGs harboring QTLs in the YX population, LGA01, A05, B02, B08, and B10 were not reported in previous studies. Therefore, 12 QTLs mapped on LGA01, A05, B02, B08, and B10 were novel. While five QTLs reported in previous works and 12 QTLs in this study were both mapped on LGA09. Similarly, 10 QTLs in the present research were mapped on LGB03, B04, and B05 which also harbored eight QTLs identified in previous study (Shirasawa et al., 2012; Huang et al., 2015, 2016; Li et al., 2017). For the XZ population, of 10 QTLs, *qPHA05.2a* on LGA05, *qPHB08.3a* on LGB08, and *qPHB10.1a* on LGB10 were reported for the first time in this study. On LGA09, LGB03, and LGB04, there were seven QTLs identified in this work and 14 QTLs reported in a previous studies (Shirasawa et al., 2012; Huang et al., 2015, 2016; Li et al., 2017). The identification of several QTLs in the present study indicated that plant height is a quantitative trait controlled by multiple genetic factors, and these novel QTLs may provide more new loci for improvement of this trait through marker-assisted selection.

Based on the above discussion, chromosome A09, B03, and B04 could stably harbor QTLs for plant height among different mapping populations. Especially for chromosome A09, almost one third of total QTLs (15/48) including eight major QTLs in two populations were located on this chromosome. These QTLs clustered together in approximately 20 cM interval in the YX population and 15 cM interval in the XZ population. These two intervals had 11 common linked markers and overlapped in the integrated map suggesting that A09 enriches genes controlling plant height. However, the order of common markers between the YX and XZ populations was not perfectly matched. Since female parent of the YX population was derived from interspecific hybridization between cultivated cultivar Baisha 1016 and a diploid wild species A. *diogoi*, heterologous genomic segments would introgress into the RIL population. Therefore, the order of several markers in the local region of A09 differed between the YX and XZ populations. To further verify that QTLs from the YX and XZ populations were co-localized on the chromosome A09, linked markers were mapped on the physical genome of *Arachis duranesis*. And a 3.4 Mb physical region on A09 that simultaneously harbor QTLs from different genetic backgrounds was identified.

Among the 161 putative genes in the region, there were five genes (*Aradu.161GD, Aradu.19EUZ, Aradu.M7AQA, Aradu.QH2NX*, and *Aradu.YUV8P*) belonging to the *FHY3/FAR1*-related gene family, which involved in phytochrome A and B signaling to control plant morphogenesis and height (Wang, 2004). In addition, two putative transcription factors, *AIL1* (*Aradu.C5HAC*) and *Brevis radix* (*Aradu.TM2EL*) were reported to take part in auxin and BR signaling, respectively, in order to regulate cell growth (Mouchel et al., 2004; Horstman et al., 2014). Additionally, there were two pectin biogenesis protein-galacturonosyltransferases (*Aradu.79NAD* and *Aradu.IVZ05*) and a cell skeleton protein-actin (*Aradu.FF624*), which are essential for cell wall formation or modification and finally affect cell elongation (Li et al., 2005; Atmodjo et al., 2011; Qin et al., 2013). However, these genes are still candidates and much work should be done to further fine map the co-localized region and verify their functions.

In conclusion, we identified 48 QTLs including 13 major QTLs for plant height in two RIL populations. Eight consistent QTLs were found to perform stably across multiple environments in the YX population. A 3.4 Mb physical interval on chromosome A09 which harbored stable QTLs from different RIL populations was also identified. It is a reliable region harboring QTLs to be further fine mapping and genes cloning. Our results provide a solid foundation for exploring the gene regulatory network of plant height, while guiding development of diagnostic makers for peanut breeding.

## Authors contributions

JL, NL, YL, LH, XZ, YC, HJ, and BL: conceived and designed the research; XR and HJ: developed two RIL populations; WC and JG: planted two RIL populations and conducted field management; NL, JL, ZX, ZL, and XL: performed the plant height management; JL: performed statistical analysis of the phenotyping data; NL: performed the QTL analysis, meta-analysis and GO annotation; NL and JL: wrote the manuscript; YL, JT, HJ, and BL: revised the manuscript. All the authors read and approved the final manuscript.

## Conflict of interest statement

The authors declare that the research was conducted in the absence of any commercial or financial relationships that could be construed as a potential conflict of interest.

## Supplementary material

Supplemental Figure 1 Distribution of QTLs on the integrated linkage group A09 from the YX population and the XZ population. The italicized and highlighted loci represent common markers between the YX and XZ populations on A09.

Supplemental Table S1 Annotation of candidate genes in a co-localized interval on chromosome A09.

Supplemental Table S2 KEGG pathway for candidate genes in a co-localized interval on chromosome A09.
