# Supplementary figures and images for: Stable QTLs for Plant Height on Chromosome A09 Identified From Two Mapping Populations in Peanut (Arachis hypogaea L.)
Source: Front Plant Sci. 2018 May 25;9:684. doi: 10.3389/fpls.2018.00684 (PMC5982159; doi:10.3389/fpls.2018.00684)

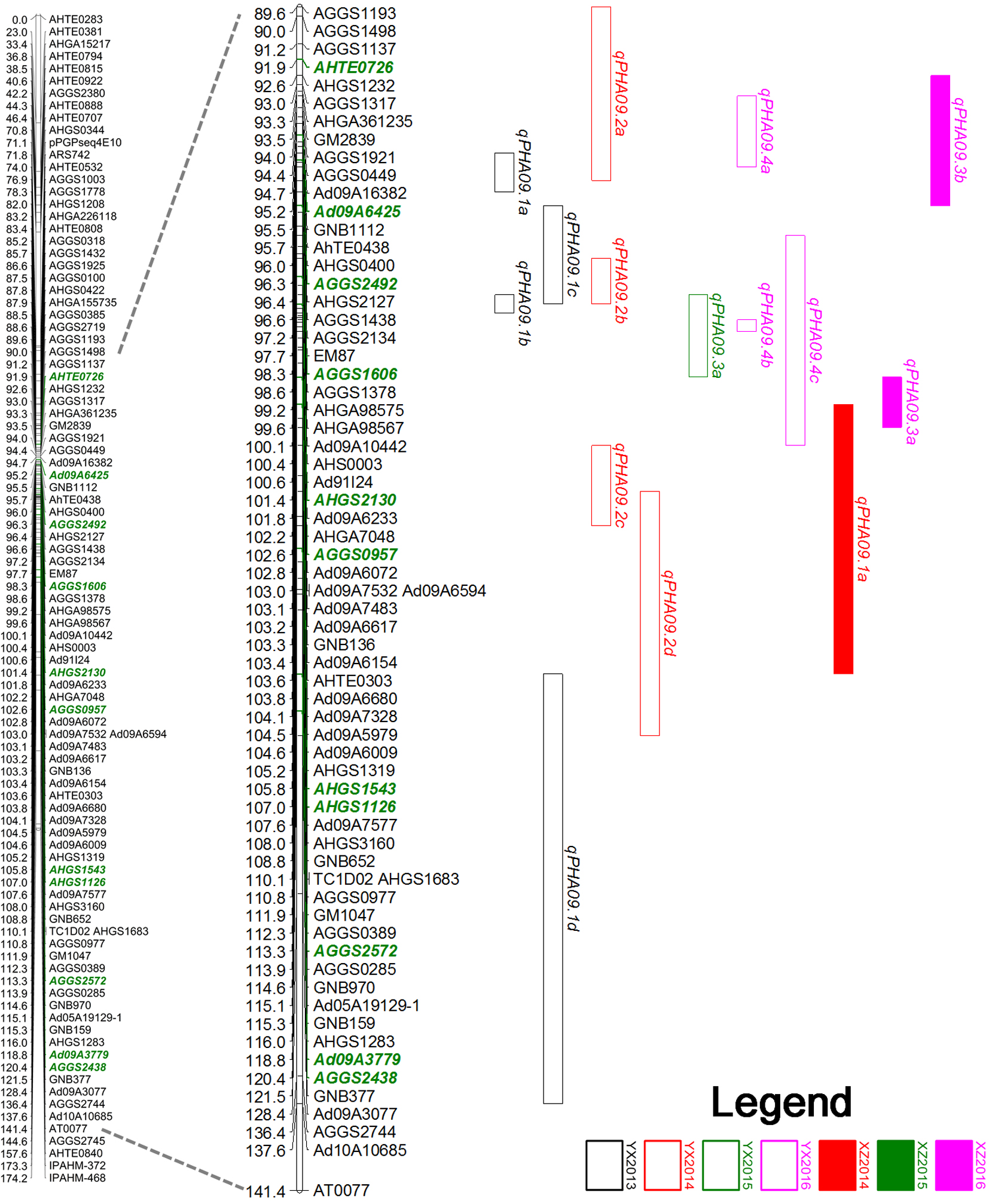

Supplement: Supplemental Figure 1 — Distribution of QTLs on the integrated linkage group A09 from the YX population and the XZ population. The italicized and highlighted loci represent common markers between the YX and XZ populations on A09. [file Image_1.JPEG]
